# Temporal Dynamics of Proactive and Reactive Motor Inhibition

**DOI:** 10.3389/fnhum.2017.00204

**Published:** 2017-04-27

**Authors:** Matthias Liebrand, Inga Pein, Elinor Tzvi, Ulrike M. Krämer

**Affiliations:** ^1^Department of Neurology, University of LübeckLübeck, Germany; ^2^Graduate School for Computing in Medicine and Life Sciences, University of LübeckLübeck, Germany; ^3^Institute of Psychology II, University of LübeckLübeck, Germany

**Keywords:** motor control, inhibition, reactive, proactive, attention, go/nogo task, oscillations

## Abstract

Proactive motor inhibition refers to endogenous preparatory mechanisms facilitating action inhibition, whereas reactive motor inhibition is considered to be a sudden stopping process triggered by external signals. Previous studies were inconclusive about the temporal dynamics of involved neurocognitive processes during proactive and reactive motor control. Using electroencephalography (EEG), we investigated the time-course of proactive and reactive inhibition, measuring event-related oscillations and event-related potentials (ERPs). Participants performed in a cued go/nogo paradigm with cues indicating whether the motor response might or might not have to be inhibited. Based on the dual mechanisms of control (DMC) framework by Braver, we investigated the role of attentional effects, motor preparation in the sensorimotor cortex and prefrontal cognitive control mechanisms, separating effects before and after target onset. In the cue-target interval, proactive motor inhibition was associated with increased attention, reflected in reduced visual alpha power and an increased contingent negative variation (CNV). At the same time, motor inhibition was modulated by reduced sensorimotor beta power. After target onset, proactive inhibition resulted in an increased N1, indicating allocation of attention towards relevant stimuli, increased prefrontal beta power and a modulation of sensorimotor mu activity. As in previous studies, reactive stopping of motor actions was associated with increased prefrontal beta power and increased sensorimotor beta activity. The results stress the relevance of attentional mechanisms for proactive inhibition and speak for different neurocognitive mechanisms being involved in the early preparation for and in later implementation of motor inhibition.

## Introduction

Motor control is one of the key executive functions in daily life, allowing us to flexibly adapt or inhibit our behavior to a changing environment according to our current goals. Two variants of inhibitory motor control can be distinguished, reactive and proactive control (Aron, 2011). Whereas reactive motor inhibition refers to a process when a prepotent movement has to be withheld or stopped in reaction to an external signal (for instance a stop sign), proactive motor inhibition describes a condition in which preparatory processes facilitate motor inhibition which might be required at a later point in time (for example a street sign warning of bears crossing).

Most behavioral and neuroscientific research on motor control has focused on reactive inhibition. However, the transferability of this model to daily life is limited as stopping usually does not happen in a purely exogenous, stimulus-driven way. Most often, context-dependent, top-down endogenous processes are involved. This for instance can be observed in psychiatric patients having deficits in urge-control, supposedly linked to a lack of such top-down signals. Proactive inhibition thus seems a more ecologically valid model and therefore promising in order to understand fundamental aspects of motor control in everyday life as well as in psychiatric and neurological disorders (for review see Aron, 2011).

The dual mechanisms of control (DMC) framework states that cognitive control can be either proactive or reactive depending on costs or benefits of the respective processes. Whereas proactive control is based upon the anticipation and prevention of interference before it occurs, reactive control relies upon the detection and resolution of interference after its onset (Braver et al., 2007; Braver, 2012). The DMC framework makes specific predictions about proactive and reactive control. During proactive control the lateral prefrontal cortex (lPFC) is believed to be activated in a sustained way, reflecting maintenance of task goals. Furthermore, attention and action are thought to be biased proactively in a goal-driven manner. Regarding reactive control, the lPFC is believed to be activated transiently in reaction to external signals. That is, according to the DMC framework three networks can be assumed to be implicated in motor control: PFC mediating cognitive control, sensorimotor cortex executing motor actions and a visuo-attention network.

In this study, investigating proactive and reactive inhibitory control, we specifically focused on these three networks. We used a cued go/nogo paradigm with cues indicating whether participants might have to stop later in response to target signals or not, thereby manipulating the need for proactive control. We took advantage of the high time-resolution of electroencephalography (EEG) and measured both event-related oscillatory activity (prefrontal beta, sensorimotor alpha/beta, occipital alpha) and event-related potentials (ERPs; contingent negative variation (CNV), N1/P1, P3). In the following paragraphs, we will outline for each of the three networks, visual-attention areas, sensorimotor cortex and PFC, what previous literature has identified as relevant ERP and oscillatory measures of reactive and proactive motor control.

In a context like the go/nogo task, in which visual targets need to be detected to successfully inhibit a preplanned response, visual attention becomes critical. In attention research, great emphasis has been put on oscillations in the alpha band (Hanslmayr et al., 2011; Klimesch, 2012) Alpha oscillations over occipital visual areas decrease during preparatory attention and increase in regions processing task irrelevant information. Moreover, visual alpha has been linked to behavioral performance in demanding tasks requiring visual attention (Ergenoglu et al., 2004; Hanslmayr et al., 2005). Lavallee et al. (2014) highlighted the role of attention in proactive motor control by showing increased delta power in trials involving proactive control, presumably reflecting the engagement of a posterior attentional network. Another marker of attentional preparation is the CNV. This slow negative potential with its maximum over central regions, is the most common ERP component when studying preparatory (i.e., proactive) cognitive processes. Schevernels et al. (2016) reported in a cued go/nogo-study that in cued nogo- compared to cued go-trials the CNV was reduced, speaking for less employment of attentional resources. Here, we expected proactive motor control to be accompanied by increased activity in attentional networks, reflected in changes in occipital alpha, the CNV and the P1/N1-complex, as ERP correlate of early visual processing in extrastriate cortex (Hillyard and Anllo-Vento, 1998).

With respect to sensorimotor cortex, oscillations in the alpha (9–13 Hz) and beta (14–25 Hz) frequency bands have frequently been associated with motor control. Sensorimotor alpha, also referred to as mu, desynchronizes during anticipation and execution of movements and synchronizes afterwards (Neuper et al., 2006). The same pattern can be observed for sensorimotor beta oscillations (Neuper et al., 2006). Previous response inhibition studies (Krämer et al., 2011; Picazio et al., 2014) have shown that beta is relatively increased during reactive motor inhibition. However, little is known about the role of sensorimotor mu and beta in proactive motor inhibition. In our study, we thus focused on mu and beta with respect to both proactive and reactive motor control.

Prefrontal regions have consistently been associated with motor inhibition (Aron et al., 2004, 2014). Moreover, prefrontal beta oscillations have been implicated in reactive motor inhibition. For instance, in healthy subjects (Alegre et al., 2004) and in epileptic patients using intracranial recordings over the right IFG (Swann et al., 2009), higher prefrontal beta power has been observed in trials where reactive inhibition was called for. Investigating proactive inhibition, two studies using a modified stop signal task (SST) in a limited sample size of epileptic patients reported increased gamma but not beta power over prefrontal electrodes (Swann et al., 2012, 2013). In the present study, we measured reactive and proactive prefrontal beta power to clarify whether proactive inhibitory control, similarly to reactive control, is reflected in increased prefrontal beta oscillations.

Finally, the most-studied ERP components of reactive response inhibition are the N2 and P3 (Huster et al., 2013). Although it remains controversial whether the stopping-related N2 and P3 reflect inhibition *per se*, recent evidence suggested that the onset of the P3, presumably stemming from PFC, is indeed linked to the success of response inhibition (Wessel and Aron, 2015). As a prominent marker of reactive inhibition, linked to the PFC, we included the P3 into our analysis.

To investigate proactive inhibition, we contrasted activity occurring in the cue-target interval, comparing trials in which subjects prepared for an upcoming inhibition with trials in which no response inhibition could occur. This comparison reflects processes modulated by the context of knowing that the default action can be implemented or that an alternative action, in this case the inhibition of an action might be required. As a second measure for proactive inhibition, we investigated how activity after target-signals was affected by the context (for a similar approach see Swann et al., 2012, 2013). Specifically, we asked how response execution was modulated by proactive control instigated by the cue. For reactive inhibition, we contrasted target-related activity in trials where subjects had to stop with trials where they executed a default motor action (for a detailed description of contrasts see “Materials and Methods” section).

Based on the DMC framework and previous findings, we expected that proactive inhibition would lead to increased employment of attention (indicated by decreased occipital alpha, an increased CNV and P1/N1), sustained higher levels of prefrontal control (reflected in increased prefrontal beta) and a modulation of sensorimotor activity (indicated by altered mu/beta power). Reactive inhibition was expected to be mediated by transiently increased prefrontal control (higher beta power), an increased P3 and increased sensorimotor mu/beta power.

## Materials and Methods

### Subjects

Twenty-five right-handed participants participated in the study. Three participants were excluded from analysis due to extensive EEG artifacts (see below for further explanation). All of the remaining 22 participants (20–32 years, mean: 24.5 years, 14 females) were by self-report free of neurological or psychiatric disorders. The participants had normal or corrected to normal vision. They gave informed consent and received money (8€/h) or course credit for participation. The study was performed in agreement with the Declaration of Helsinki and had been approved by the ethics committee of the University of Lübeck.

### Design and Stimuli

**Figure 1:**
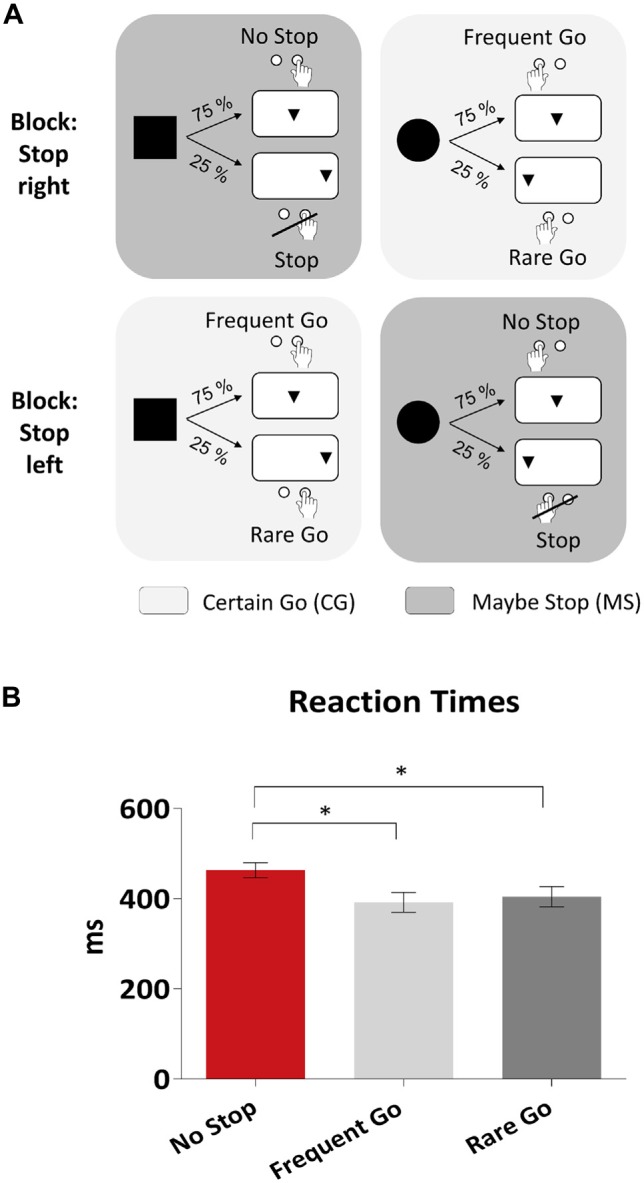
**Design and behavioral results. (A)** Design of the cued go/nogo task. On the upper row a block is illustrated where subjects had to refrain from pressing the button when the target (triangle) appeared on the right side of the screen. Similarly, in the block displayed in the lower row a stop was required when the target appeared on the left side. In the **Maybe Stop (MS)** condition (dark gray) the subject was told by the cue (square, circle) that he might have had to stop later on, while in the **Certain Go (CG)** condition (light gray) he was informed by the cue that he always could press the button when the target appeared later on. **(B)** Behavioral results showing mean reaction times in No Stop-, Frequent Go- and Rare Go-trials. As error bars the standard errors of the mean (SEM) are displayed. Significant effects are stressed with asterisks.

The participants performed a cued go/nogo-task (see Figure 1). The cue-stimuli were a square and a circle, the following target stimulus a triangle. If the triangle followed the square, it was in 75% of the trials presented in the center of the screen and in 25% lateralized to the right side (5°). If the triangle followed the circle, it appeared in 75% of the trials in the center of the screen and in 25% lateralized to the left side (5°). The probability of the two cues was 50% each.

The participants were instructed to press the right button (go) with their right index finger after the triangle following the square and the left button with their left index finger after the triangle following the circle. Additionally, before each block they were told to refrain from pressing the button either when the triangle appeared on the right side (block: stop right) or on the left side (block: stop left). These two types of trials were alternated from block to block. Participants were instructed before each block which side they have to attend and possibly stop.

This design results in two different conditions, which we refer to as Maybe Stop (MS) and Certain Go (CG; see Figure 1). In the MS condition, the cue indicated that the participant might have to stop afterwards, whereas in a CG condition no stop was required in any case. In the MS condition participants had to stop in 25% of the trials (Stop-trials), but were instructed to press the button in the remaining 75% (No Stop-trials). In terms of probability and visual stimuli the trials in the CG condition were matched to Stop- and No-Stop-trials. Trials in the CG condition which are matched to Stop-trials, thus including lateralized target stimuli and 25% probability, are referred to as Rare Go-trials. The remaining 75% of trials, matched to No Stop-trials, are referred to as Frequent Go-trials. For instance, if the current block was a “stop-left” block and a circle was presented followed by a triangle on the left side, the response had to be withheld (MS condition, Stop-trial). However, if in the same block a square was presented followed by a triangle on the right side, the participant had to press the right button (CG condition, Rare Go-trial). Also, in the following we refer to trials with 75% probability as frequent trials (No Stop and Frequent Go) and to those with 25% probability as rare trials (Stop, Rare Go).

Both cue (square, circle) and target (triangle) appeared for 100 ms. The target followed cue-onset after an interval of 1100 ms. The time between two subsequent trials was jittered (1.3–1.6 s). The experiment was divided into six blocks with 160 trials each, resulting in 960 trials. The to-be-stopped side of the first block was counterbalanced among participants. Throughout the whole experiment a fixation line was presented underneath the stimuli, which participants were instructed to fixate. Participants were instructed to respond as fast and accurate as possible and not to press the button until the triangle had appeared. Before the start of the experiment the participants practiced the task in three short blocks with each 16 trials.

### Procedure

The experiment was controlled using the Presentation® software (Version 14.5). Stimuli were presented on a 17″ screen, about 1 m away from the participant. Participants were sitting in a comfortable chair and in the middle and after each of the experimental blocks they had a short break of 20 s where they were able to relax. The total duration of the experiment was about 50 min.

### EEG Recordings and Data Preprocessing

The EEG was recorded with a 64-channel BrainAmp MR plus amplifier with a sampling rate of 250 Hz. Electrodes were placed according to an extension of the international 10–20 system (Nuwer et al., 1998). Vertical and horizontal eye movements (vEOG and hEOG) were recorded, the former using an electrode placed below the right eye and a frontopolar electrode, the latter using electrodes located on the outer canthus of each eye. The EEG was recorded against a reference electrode placed on the right earlobe.

### Behavioral Data Analyses

Mean reaction times, overall error rates and premature error rates (button presses before the triangle had appeared) were computed for each subject and submitted to paired sample *t*-tests comparing Stop-, No Stop-, Frequent Go- and Rare Go-trials.

### EEG Data Analyses

EEG data analysis was performed with EEGLAB (Delorme and Makeig, 2004), ERPLAB (Lopez-Calderon and Luck, 2014) and custom written MATLAB (Natick, MA, USA) scripts. EEG data were re-referenced offline to the average of the signal from the two earlobe electrodes. The data were high-pass filtered with 0.5 Hz in addition to a notch filter of 50 Hz. The data were segmented into epochs for the different conditions. Epochs included 1 s before and 2 s after the stimulus. The baseline was defined as the 100 ms preceding the stimulus, with the stimulus being either the cue or the target, depending if effects in the cue-target interval or target-related activity was analyzed. An Independent Components Analysis (ICA), as implemented in EEGLAB (Infomax extended), was performed on the epoched data including all conditions. Independent components accounting for blink artifacts and horizontal eye movements were identified and removed from the data (Jung et al., 2000). Trials affected by other artifacts caused, e.g. by muscle tension, were rejected from further analysis with a threshold for rejection of ±80 μV. If more than 30% of the data of one participant were rejected, this subject was excluded from analysis (three subjects). Current source density (CSD) interpolation of the data was estimated through Laplacian computation based on a spherical spline interpolation (with a spline order of four; Kayser and Tenke, 2006) using a toolbox for MATLAB (Kayser, 2009). We took advantage of the Laplace transformation, as it improves the spatial resolution of EEG, especially in combination with higher density recordings (≥64 electrodes; Babiloni et al., 1995).

#### Event-Related Potentials (ERPs)

We analyzed the amplitude of the CNV in the cue-target interval and the target-related P1, N1 and P3. As measure of the amplitude, we computed the area under the curve (AUC) in a given time-window, zeroing negative values in positive waveforms and vice versa. The AUC is a rather new approach but has the critical advantage of minimizing the problem of selecting an appropriate measurement window (Luck, 2014). Also, when computing our statistics based on mean amplitude measures, all reported significant effects were replicated. P1 and N1 were measured at PO7 and PO8 and in the following time-windows after target-onset: P1 (50–150 ms), N1 (100–250 ms). The CNV was measured in an interval directly preceding the target (−100 to 0 ms relative to the target), as its amplitude is expected to be more stable later during a preparatory process (Boehm et al., 2014) and most representative of the preparatory state directly before target-onset. The CNV was measured at Cz, as this slow negativity has been shown to be highest over central sides in a motor preparation context (Filipović et al., 2001; Smith et al., 2013). P3 was measured between 300 ms and 500 ms after target-onset, a common time-range for P3 in go/nogo tasks (Verleger et al., 2006; Smith et al., 2008). We decided to measure P3 at central midline electrodes (C3, C4, Cz). This decision was data-driven and based on the topography of the go-P3 (Figure 5), which had two lateralized maxima over the sensorimotor cortex. This topography is likely due to application of CSD transformation to our data, as data without transformation show a central topography. For P1 and N1, data of trials requiring left-handed responses were flipped along the midline to be able to measure activity over the visual cortex contra- and ipsilateral to the relevant stimulus side. That is, we analyzed effects in the contra- and ipsilateral hemisphere to lateralized target-stimuli. For all components, data were averaged across trials with right- and left-hand responses. Data of the early components P1 and N1 were subjected to repeated measures ANOVAs with the within-subject factors Condition (MS vs. CG) and Hemisphere (ipsi- vs. contralateral to the lateralized target stimulus). Data of target-evoked P3 were subjected to repeated measures ANOVAs with the within-subject factors Condition (MS vs. CG) and Electrode (C3, Cz, C4). The CNV was analyzed with a paired sample *t*-test comparing MS- and CG-trials. For visualization only, data were low-pass filtered with 15 Hz.

**Figure 2:**
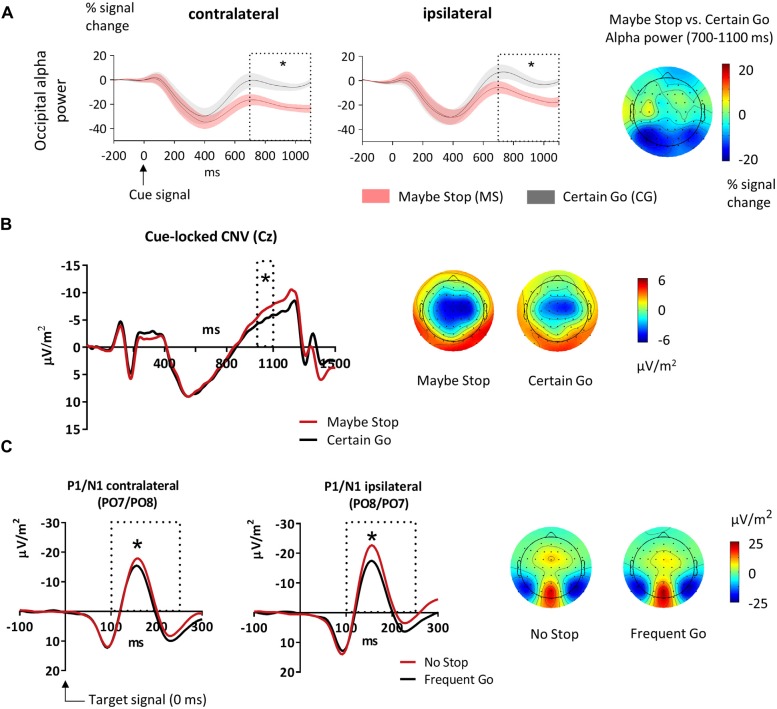
**Effects of attentional gating. (A)** Alpha (9–14 Hz) power in the cue-target interval for the Maybe Stop (red) and the Certain Go condition (black) at occipital clusters. The analyzed time-window (700–1100 ms) is displayed as dotted box. The cue appeared at 0 ms and the target stimulus at 1100 ms. Alpha power was lower in MS- than CG-trials both contralateral and ipsilateral to the upcoming, possibly lateralized target. The SEM is displayed as shaded area. The topographic plot to the right shows the scalp distribution of the alpha band as difference between Maybe Stop- and Certain Go-trials. **(B)** Contingent negative variation (CNV) in the cue-target interval, measured at Cz. Displayed are MS- (red) and CG-trials (black). In the analyzed time-window (1000–1100 ms, dotted box) the CNV was increased in MS- compared to CG-trials. The target stimulus appeared at 1100 ms. The topographic plots display mean activity between 1000 ms and 1100 ms. **(C)** Target-evoked P1/N1 at sites contra- and ipsilateral to the visual stimuli. In the analyzed time-window (100–250 ms, dotted box) N1 was increased for No Stop- than Frequent Go-trials. The topographies display mean activity between 130–170 ms. Here 0 ms indicates time of target-onset. Significant effects are stressed with asterisks.

**Figure 3:**
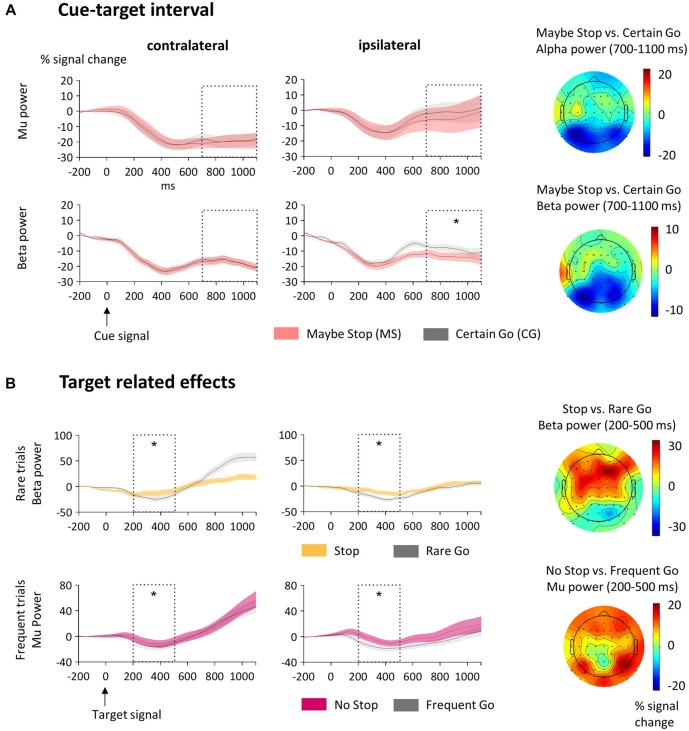
**Sensorimotor activity. (A)** Activity in the cue-target interval. Here mu (9–14 Hz; upper row) and beta (15–25 Hz; lower row) power at sensorimotor clusters contralateral and ipsilateral to the standard motor response is displayed. The analyzed time-window (700–1100 ms) is indicated as dotted box and the SEM is shown as shaded area. The cue appeared at 0 ms and the target stimulus at 1100 ms. Mu was lower on the contralateral than on the ipsilateral side but did not differ between MS- and CG-trials. Beta decreased in MS-trials on the ipsilateral side only. The topographic plots to the right show the scalp distribution of the alpha and beta bands as differences between MS- and CG-trials. **(B)** Target-related effects of sensorimotor mu and beta. The analyzed time-window (200–500 ms) is indicated as dotted box. Here 0 ms indicates time of target-onset. Upper row: Beta power was higher in Stop- than Rare Go-trials at both sensorimotor clusters contralateral and ipsilateral to the motor response. Lower row: Mu power was higher in No Stop- than Frequent Go-trials at contralateral and ipsilateral sensorimotor clusters. The topographic plots to the right show the scalp distribution of the alpha and beta bands as differences between Stop- and Rare Go-, or respective No Stop- and Frequent Go-trials. Significant effects are stressed with asterisks.

**Figure 4:**
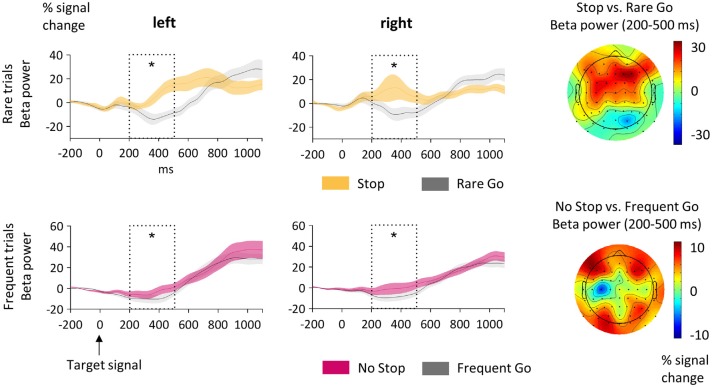
**Target related prefrontal activity**. Here beta (15–25 Hz) power at left and right prefrontal clusters is displayed. The analyzed time-window (200–500 ms) is indicated as dotted box and the SEM is shown as shaded area. Beta was higher in Stop- than Rare Go-(upper row) and in No Stop- than Frequent Go-trials (lower row). The topographic plots to the right show the scalp distribution of the alpha and beta bands as differences between Stop- and Rare Go- or respective No Stop- and Frequent Go-trials. Significant effects are stressed with asterisks.

**Figure 5:**
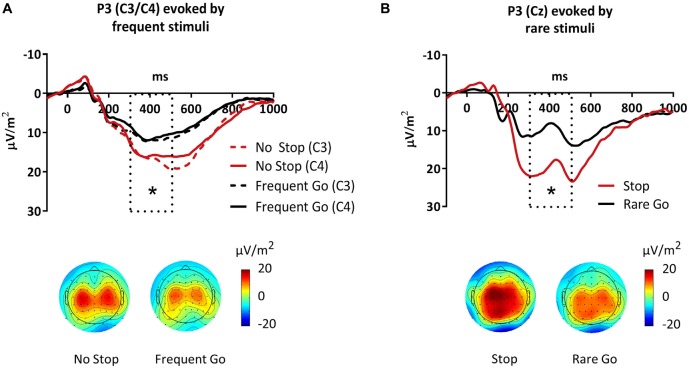
**P3 evoked by frequent and rare target-stimuli**. The analyzed time-window (300–500 ms) is shown as dotted box. The topographies display mean activity between 300 ms and 500 ms in the different conditions. **(A)** P3 evoked by frequent stimuli was increased in No Stop- compared to Frequent Go-trials at both C3 and C4. **(B)** P3 evoked by rare stimuli was increased in Stop- compared to Rare Go-trials at Cz. Significant effects are stressed with asterisks.

#### Time-Frequency Analysis

To study the inhibition related power changes in the alpha and beta band, single trial data were convolved with a complex Morlet wavelet as implemented in MATLAB (function cwt with parameter specification “cmor1–1.5”):

$$w(t)={\left(\pi f_{b}\right)}^{-0.5}e^{-2\pi ift_{c}}e^{-\frac{t^{2}}{f_{b}}}$$

where *f*_b_ = 1 was the bandwidth parameter and *f*_c_ = 1.5 was the wavelet center frequency (Teolis, 1998). Specifically, we computed and averaged for each subject changes in time varying energy (square of the convolution between wavelet and signal) in the studied frequencies (1–40 Hz, linear increase) with respect to a pre-stimulus baseline (−250 ms to −50 ms prior to the stimulus). The selection of the analyzed alpha/mu (9–14 Hz) and beta (15–25 Hz) frequencies was based on visual inspection of the data and previous literature (Krämer et al., 2011; Solbakk et al., 2014). In order to reduce the number of statistical comparisons and to increase signal-to-noise ratio, we clustered the electrodes into regions of interest: Left prefrontal (F3, F5, FC3, FC5), right prefrontal (F4, F6, FC4, FC6), left central (C3, C5, CP3, CP5), right central (C4, C6, CP4, CP6), left parieto-occipital (P5, P7, PO3, PO7) and right parieto-occipital (P6, P8, PO4, PO8) based on a previous study (Krämer et al., 2013). Effects over prefrontal, sensorimotor and occipital electrodes were analyzed differently. For motor related (sensorimotor mu and beta) and attention-related (occipital alpha) effects, data of trials requiring a left hand response were flipped along the midline (for a similar approach see e.g., Fogelson et al., 2009). In the motor- and visual networks, lateralized activity could be expected as unimanual responses were given and lateralized stimuli were presented. Data was flipped along the midline to average across responses with the right/left hand and over lateralized presented stimuli. We were thereby able to analyze effects in the hemispheres contra- and ipsilateral to the respective response hand or visual stimulus. For prefrontal effects (beta over prefrontal electrodes) data was not flipped, because we did not expect these effects to be lateralized dependent on the side of motor actions or visual stimuli. That is, we compared data of left and right hemisphere. For all effects, data of trials with right- and left-hand responses were averaged. Then mean time-frequency power in a given time-window (see below) was subjected to repeated measures ANOVAs with the within-subject factors Condition (MS vs. CG) and Hemisphere (ipsi- vs. contralateral or left- vs. right). As measurement window for effects before target onset we chose 700–1100 ms because the cue-related activity was distant and target-onset (1100 ms) was closest. For target-related effects we investigated the window between 200 and 500 ms. For stopping-related effects of beta power over prefrontal electrodes similar time-windows have been reported before (Swann et al., 2009, 2011) and this timeframe encloses the period when participants enacted or inhibited their motor response after having processed the cue-stimulus. To account for multiple comparisons that were performed for each individual condition difference, we applied corrected *p*-values using the Bonferroni method (see “Results” section).

As a measure for proactive inhibition in the cue-target interval, we contrasted trials in which the later motor response might (MS) or might not have to be inhibited (CG). To assess how proactive inhibition modulated response execution after target signals, we compared trials following cues that signaled that responses might (No Stop) or might not have to be stopped (Frequent Go; see Swann et al., 2012, 2013). Finally, to assess the correlates of reactive inhibition, we contrasted trials in which the response actually had to be inhibited (Stop) with matched go-trials still requiring a response (Rare Go).

## Results

### Behavioral Results

Participants were faster in Frequent- and Rare Go- compared to No Stop-trials (Figure 1; Frequent Go- (392 ± 103 ms) vs. No Stop- (463 ± 79 ms) trials: *t*_21_ = 8.0, *p* < 0.001; Rare Go- (404 ± 105 ms) vs. No Stop-trials: *t*_21_ = 4.4, *p* < 0.001). Subjects responded more accurately in No Stop- (98.0%) than Frequent Go-trials (95.1%; *t*_21_ = 2.1, *p* = 0.044), due to a higher rate of premature errors (button presses before the triangle had appeared) in Frequent Go-trials (*t*_21_ = 2.4, *p* = 0.027). Also, in Rare Go-trials more premature errors than in Stop-trials were committed (*t*_21_ = 2.2, *p* = 0.037).

### EEG Results

As we performed several analyses in the time-frequency domain, we used Bonferroni corrected *p*-values, separately for effects taking place before and after target stimuli. In the cue-target interval, we conducted four comparisons (alpha at occipital regions, mu/beta at sensorimotor regions and beta at prefrontal regions) which results in a corrected *p*-value of 0.0125. For target-related effects six comparisons were conducted (beta at prefrontal regions for frequent and rare-trials and mu/beta at sensorimotor regions for frequent and rare-trials). This results in a corrected *p*-value of 0.0083. For main findings of this study see Table 1.

**Table 1:** **Summary of main findings**.

	Proactive	Reactive
**Attention**
*Occipital alpha*	↓
*N1/P1*	After target onset: ↑	−
*Proactive motor control led to increased attention in the Maybe Stop condition where informative target stimuli were anticipated and presented*.		
**Sensorimotor effects**
*Mu*	Before target onset: -
	After target onset: ↑	(↑)
Beta	Before target onset: ↓ IL side
	After target onset: -	↑
*Before target onset, only beta was modulated by proactive inhibition but not mu. After target signals, mu and not beta was increased for proactive inhibition. Both mu and beta increased for reactive inhibition*.		
**Prefrontal effects**
*Prefrontal beta*	Before target onset: -
	After target onset: ↑	↑
*Proactive and reactive motor inhibition were modulated by increased prefrontal control. Prefrontal activity for proactive inhibition occurred only after target onset* *but not before. Prefrontal areas were thus activated transiently rather than in a sustained way*.		

*IL = ipsilateral, ↓ = decrease, ↑ = increase, - = no effect*.

#### Visual Attention Effects

##### Occipital alpha

To investigate the role of visual attention in proactive motor control, we examined condition differences in alpha power over occipital sites in the cue-target interval. After cue-onset in both Maybe Stop- and Certain Go-trials, alpha decreased until about 400 ms and then increased again. Whereas alpha in CG-trials increased towards the baseline level, this rebound was dampened in MS-trials resulting in reduced alpha power (Figure 2A). We subjected mean alpha power between 700 ms and 1100 ms to repeated measures ANOVAs with the factors Condition (MS, CG) and Hemisphere (contra-, vs. ipsilateral to the upcoming, possibly lateralized target stimulus). Alpha power was lower in MS- compared to CG-trials (*F*_(1,21)_ = 22.5, *p* < 0.001). Also, alpha activity was lower in the hemisphere contralateral to the upcoming, possibly lateralized stimulus, compared to ipsilateral (*F*_(1,21)_ = 19.9, *p* < 0.001), but the two factors did not interact (*F*_(1,21)_ = 1.4, *p* = 0.245). For time-frequency plots of attentional as well as sensorimotor and prefrontal effects see Supplementary Figure S1.

##### CNV

The CNV, measured in the cue-target interval, had a typical central topography, with a maximum at Cz and increasing towards the target (Figure 2B). We subjected the AUC between 1000 ms and 1100 ms to a paired sample *t*-test comparing MS- and CG-trials. The CNV was larger in MS-trials compared to CG-trials (*t*_21_ = 2.5, *p* = 0.021).

##### Target-related P1/N1

To assess attention related effects on early visual target processing, we looked at the P1/N1 complex to the target stimuli. P1 peaked between 90–130 ms and N1 around 150–200 ms. Around the time of their maxima, both components were centered at PO7 and PO8 (Figure 2C). We subjected the AUC between 50–150 ms (P1) and 100–250 ms (N1) to ANOVAs with the factors Hemisphere (ipsi-, vs. contralateral to the target stimulus) and Condition (frequent trials: No Stop vs. Frequent Go, rare trials: Stop vs. Rare Go).

The N1 elicited by centrally presented stimuli was larger in No Stop- than Frequent Go-trials (*F*_(1,21)_ = 11.7, *p* = 0.003). The P1 did not differ between No Stop- and Frequent Go-trials (*F*_(1,21)_ = 1.2, *p* = 0.288). In trials with lateralized target-stimuli (Stop vs. Rare Go), neither N1 nor P1 showed a difference for the factor Condition (N1: *F*_(1,21)_ = 0.7, *p* = 0.422; P1: *F*_(1,21)_ = 0.3, *p* = 0.611).

#### Sensorimotor Effects

First, we were interested in the modulation of mu and beta activity over the sensorimotor cortex in preparation of the upcoming motor action (Figure 3A). That is, in the cue-target interval mean mu and beta power between 700 ms and 1100 ms was subjected to an ANOVA with the factors Condition (MS vs. CG) and Hemisphere (contra-, and ipsilateral to the upcoming motor response). In the beta band, power in the hemisphere ipsilateral to the relevant response hand was lower in MS- compared to CG-trials (Condition × Hemisphere: *F*_(1,21)_ = 11.6, *p* = 0.003; Condition at the ipsilateral hemisphere: *t*_21_ = −4.3, *p* < 0.001; Condition at the contralateral hemisphere: *t*_21_ = −0.2, *p* = 0.871). Beta power was also found to be generally reduced in the contralateral relative to the ipsilateral hemisphere (Hemisphere: *F*_(1,21)_ = 13.7, *p* = 0.001). In the mu band there was no difference between MS- and CG-trials (*F*_(1,21)_ = 1.0, *p* = 0.319). However, mu was lower on the contralateral than on the ipsilateral side (*F*_(1,21)_ = 11.8, *p* = 0.002).

Next, we tested how sensorimotor mu and beta power were modulated by proactive and reactive motor control in the timeframe after target signals appeared (Figure 3B). Therefore we subjected target-related mean mu/beta power around the time of response execution (200–500 ms) to ANOVAs with the factors Condition (frequent trials: No Stop vs. Frequent Go, rare trials: Stop vs. Rare Go) and Hemisphere (ipsi- vs. contralateral to the motor response). In rare trials (Stop vs. Rare Go), sensorimotor beta power was higher in Stop- than Rare Go-trials (*F*_(1,21)_ = 20.5, *p* < 0.001) and also activity in the mu band tended to be higher in Stop- than Rare Go-trials (*F*_(1,21)_ = 3.6, *p* = 0.072). Comparing frequent trials (No Stop vs. Frequent Go), sensorimotor mu power was higher in No Stop- than in Frequent Go-trials (*F*_(1,21)_ = 12.2, *p* = 0.002), but there were no condition differences in the beta band (*F*_(1,21)_ = 0.0, *p* = 0.972).

#### Prefrontal Activity and P3

##### Prefrontal activity

First, we analyzed whether beta power over prefrontal electrodes was modulated in the cue-target interval. We therefore subjected mean beta power at prefrontal sites between 700 ms and 1100 ms to an ANOVA with the factors Condition (MS vs. CG) and Hemisphere (left vs. right). Prefrontal beta did not differ between conditions (*F*_(1,21)_ = 0.2, *p* = 0.643). This can also be assessed from the topographic map shown in Figure 3A which shows no differential beta power over prefrontal sites (lower topographic plot).

To test for a modulation of beta power after target signals, we subjected mean beta power over prefrontal electrodes between 200 ms and 500 ms to repeated measures ANOVAs with the factors Condition (MS vs. CG) and Hemisphere (left vs. right; Figure 4). This was done separately for rare (Stop- vs. Rare Go-) and frequent (No Stop- vs. Frequent Go-) trials. Beta power over prefrontal electrodes was higher in Stop- than Rare Go-trials (*F*_(1,20)_ = 8.9, *p* = 0.007). Note that one participant showed extreme beta power compared to other subjects (>10 SD above mean value) and therefore was removed from the statistics regarding this comparison. Proactive beta power was also increased in No Stop- compared to Frequent Go-trials (*F*_(1,21)_ = 10.2, *p* = 0.004). The effect did not differ between hemispheres in either rare or frequent trials (Condition × Hemisphere for rare trials: *F*_(1,20)_ = 0.1, *p* = 0.772 and for frequent trials: *F*_(1,21)_ = 0.3, *p* = 0.571).

To test whether after target signals the effect of Condition on beta differed between frequent and rare trials, we computed an ANOVA including the factors Frequency (rare, frequent), Condition (MS, CG) and Hemisphere. Beta power was higher in rare than frequent trials (*F*_(1,20)_ = 12.2, *p* = 0.002) and the two types of trials differed in the MS condition but not in the CG condition (Frequency × Condition: *F*_(1,20)_ = 5.5, *p* = 0.03; Frequency for MS: *F*_(1,20)_ = 9.2, *p* = 0.007; Frequency for CG: *F*_(1,20)_ = 0.2, *p* = 0.641), reflecting an increased beta response in Stop- relative to No Stop-trials but no difference between Rare Go- and Frequent Go-trials. Please note that we did not specifically control for frontal muscular artifacts. Thus these might have affected our data. Consider however, that muscular artifacts should not be condition specific and therefore unlikely contribute to reported effects and very similar results have been obtained in a study using electrocorticography (ECoG; Swann et al., 2009).

##### P3

Finally, we investigated the target-evoked P3. The P3 in frequent trials (No Stop, Frequent Go) was maximal around 400 ms and, in contrast to the typically reported central maximum, it showed two lateralized maxima at C3 and C4 (Figure 5). The P3 in rare trials (Stop, Rare Go) however, had a more central maximum. In both frequent and rare trials, we verified the maximum over central electrodes by testing three clusters of electrodes (F3, Fz, F4—C3, Cz, C4—P3, Pz, P4), revealing that it was larger over central than prefrontal and parietal sites (Site: *F*_(1,21)_ = 15.3, *p* < 0.001). The P3 elicited by frequent stimuli was larger in No Stop- compared to Frequent Go-trials (*F*_(1,21)_ = 11.4, *p* = 0.003). This effect was larger over C3 and C4 than Cz (Condition × Electrode: *F*_(1,21)_ = 6.6, *p* = 0.004; Condition at C3: *t*_21_ = 3.5, *p* = 0.002; Condition at C4: *t*_21_ = 4.5, *p* < 0.001; Condition at Cz: *t*_21_ = 1.2, *p* = 0.260).

The P3 in rare trials had a less focused topography than in frequent trials. It was larger in Stop- compared to Rare Go-trials (*F*_(1,21)_ = 20.7, *p* < 0.001). Here, this effect was larger over Cz than C3 and C4 (Condition × Electrode: *F*_(1,21)_ = 5.0, *p* = 0.012; Condition at Cz: *t*_21_ = 4.3, *p* < 0.001; Condition at C3: *t*_21_ = 3.6, *p* = 0.002; Condition at C4: *t*_21_ = 2.1, *p* = 0.047). Thus, the target-evoked P3 was increased when participants had anticipated a possible stop in the cue-target interval (MS) before, compared to CG-trials, both for rare and frequent signals. To test whether this effect was identical for rare and frequent stimuli, over all tested electrodes (C3, Cz, C4) and whether there was a specific effect of stopping, we also computed an ANOVA including the factors Condition (MS vs. CG), Frequency (rare trials vs. frequent trials) and Electrode (C3, Cz, C4). The P3 was larger in trials where in the cue-target interval participants had anticipated a possible stop (MS) than in CG-trials (*F*_(1,21)_ = 19.8, *p* < 0.001) and larger in rare compared to frequent trials (*F*_(1,21)_ = 20.1, *p* < 0.001). An interaction of the factors Condition, Frequency and Electrode showed that the effect of Condition in frequent trials was largest over C3 and C4 whereas in rare trials it was largest over Cz (Condition × Frequency × Electrode: *F*_(1,21)_ = 13.2, *p* < 0.001).

## Discussion

We investigated the temporal dynamics of proactive and reactive motor inhibition in a cued go/nogo task. During the cue-target interval, when participants prepared to either respond to the target or to possibly inhibit the motor response in case of MS-trials, proactive motor inhibition was associated with decreased occipital alpha power reflecting increased visual attention. Further supporting the implication of attention in proactive motor control, the CNV was found to be increased during this time and the target-evoked N1 was enhanced. Moreover, sensorimotor beta power ipsilateral to the prepared response hand was decreased in MS-trials. After the target, proactive and reactive motor control were reflected in increased beta power over prefrontal electrodes and in an increase of P3. Our results emphasize the importance of attention for proactive motor control and demonstrate that proactive inhibition modulates ipsilateral sensorimotor activity. Prefrontal control, as reflected in beta activity, was found to be employed in a phasic manner after target onset only, but not during the preparatory cue-target interval (see main findings in Table 1).

As predicted by the DMC theory, proactive motor inhibition was associated with increased attention and a modulation of sensorimotor and prefrontal activity. However prefrontal activity was not elicited in a sustained manner before target onset, but only transiently after target signals. This is against the predictions of the DMC, but dovetails recent findings in other motor inhibition paradigms (Swann et al., 2012, 2013; Zandbelt et al., 2013; Vink et al., 2015). In the following, we will first discuss behavioral results and then refer to results pertaining to the different aspects of proactive and reactive control implied in the DMC model, namely visual attention, prefrontal control and sensorimotor activity.

### Behavioral Data

In No Stop-trials, when participants were prepared to withhold their motor response, they slowed down in comparison to Frequent Go- and Rare Go-trials when stopping was not required. This slowing is in line with previous work (Verbruggen and Logan, 2008; Jahfari et al., 2012; Zandbelt et al., 2013; Vink et al., 2015). Moreover, participants committed more premature errors during the CG than during in the MS condition. Together, behavioral data show that the paradigm was successful in eliciting proactive inhibition, leading to slower responses when stopping was anticipated.

### Visual Attentional Gating

In the interval between cue and target, oscillatory alpha power over the occipital cortex showed a stronger reduction in MS than in CG-trials. Alpha activity over occipital sites has been interpreted as a gating mechanism, meaning that an increase in alpha reflects a suppression whereas a decrease facilitates processing of incoming visual input (Romei et al., 2010; Foxe and Snyder, 2011; Zumer et al., 2014). We thus believe that the decrease of alpha activity prior to target onset reflects enhanced allocation of attentional resources since in MS-trials the location of the upcoming target was of task relevance whereas in CG-trials this was not the case. Increased visual attention in the MS compared to the CG condition should result in enhanced processing of target stimuli in No Stop- and Stop-trials, measurable as increased N1 (Luck et al., 2000). This is exactly what we found as in No Stop-trials, the visual N1 was greater than in Frequent Go-trials. This was not observed for rare signals (Stop vs. Rare Go) though, but as these stimuli were also less frequent, the higher saliency might have led already to an increase. Our results support previous research showing the relevance of attentional processes in proactive inhibition. A recent behavioral study stressed that proactive (and reactive) motor control strongly rely on attentional and perceptual processes (Verbruggen et al., 2014). Lavallee et al. (2014) observed increased delta power over posterior electrodes during proactive control, presumably reflecting activity of a posterior attentional network. Finally, our results dovetail with those of Schevernels et al. (2016), who reported a higher N1 to task-relevant go-targets compared to task-irrelevant nogo-targets.

Further evidence for the implication of attentional networks in proactive motor control was given by the CNV. The late CNV is thought to be a neural correlate of anticipatory attention towards the upcoming stimulus and of motor preparation (Tecce, 1972; Brunia and Van Boxtel, 2001). In agreement with this, the late CNV is presumably comprised of at least two slow waves, namely a stimulus-preceding negativity (SPN) and a movement preceding negativity (MPN; Brunia, 1988). The larger CNV in the MS compared to the CG condition can be explained with stronger need for attentional resources and higher expectancy of relevant information (Fuentemilla et al., 2013). However, it cannot be ruled out that also increased preparatory motor processes caused or contributed to this effect.

### Sensorimotor Activity

In the cue-target interval, we expected oscillations in the sensorimotor cortex as index of motor preparation to be influenced by proactive motor inhibition. Over sensorimotor electrodes, we observed a stronger decrease of mu power contra- than ipsilateral to the response hand. This is in line with findings that mu decreases in anticipation of movements (Babiloni et al., 2004; Kajihara et al., 2015; Tzagarakis et al., 2015) and that the decrease is most prominent over the contralateral hemisphere of the expected movement (Neuper et al., 2006). However, we did not find differences in mu between MS- and CG-trials. On the other hand, we observed sensorimotor beta in the ipsilateral hemisphere to be lower in MS- compared to CG-trials, whereas no differences were found over contralateral sites. A possible, yet speculative explanation is that a relative increase in beta oscillations over the ipsilateral motor cortex could facilitate response activation in the contralateral cortex via interhemispheric connections. This might be dampened when expecting a nogo-signal, leading to a reduced lateralization of sensorimotor beta. A study using transcranial magnetic stimulation (TMS) points in this direction, showing that the left premotor cortex is involved in withholding and releasing a preselected movement generated by the right motor cortex (Kroeger et al., 2010). Future studies could directly test this hypothesis by combining double-pulse TMS protocols with EEG.

After target-onset, mu activity decreased more in trials of the CG condition compared to the MS condition. Since the observed mu decrease was maximal around the time of button press and response times in CG-trials were considerably faster than in MS-trials, this effect fits with a role of mu for gating functions of the sensorimotor cortex (for review see Cheyne, 2013). Proactive inhibition thus resulted in a modulation of target-evoked response preparation. With respect to actual stopping of motor output, we observed lower beta power in trials where subjects pressed a button compared to trials in which they inhibited motor behavior. Such a movement related beta decrease has been observed in numerous studies (Neuper et al., 2006; Zaepffel et al., 2013), mostly centered on the sensorimotor cortex with a contralateral predominance (Salmelin and Hari, 1994; Taniguchi et al., 2000). A relative increase of sensorimotor beta has also previously been reported for stop-trials in a stop-signal task (Krämer et al., 2011), which is in line with the observed beta increase when participants inhibited the motor output.

### Dynamics of Prefrontal Activity

Reactive motor inhibition has previously been hypothesized to be driven by beta-oscillations in a frontal-basal-ganglia network (Aron, 2011). However, less is known about the role of prefrontal beta oscillations in proactive motor inhibition. With the present study, we show that prefrontal beta power is increased during the proactive and reactive implementation of response inhibition, that is, after the target, but not while preparing to stop during the cue-target interval. This dovetails with two recent fMRI-studies which compared cue-target and target-interval and reported the rIFG to be activated after, but not before the target (Zandbelt et al., 2013; Vink et al., 2015). Also in two ECoG-studies, gamma activity over prefrontal regions was higher after target-onset only, but not in the cue-target interval (Swann et al., 2012, 2013). Together, these results and our findings speak for a specific time-course of activity in prefrontal regions during proactive motor control. According to the DMC theory sustained activation of prefrontal regions can be expected for proactive control. Our data do not support this prediction, since no correlates of sustained PFC activity were detected before appearance of the target. Only during actual response execution or inhibition, PFC activity was observed. Taking prefrontal beta as correlate of cognitive control, our data suggest that activity in perceptual and sensorimotor regions is biased without sustained input from PFC. The PFC rather transiently exerts control once response conflicts occur and predominant responses have to withhold.

Interestingly, prefrontal beta power was not only increased in Stop-trials, meaning during actual response cancellation. Beta power was also higher in No Stop-trials, in which participants had been prepared to stop the response, in comparison to Frequent Go-trials, in which the action could be executed in a rather automatic fashion. Prefrontal beta thus does not simply signal an action to be canceled, as previously hypothesized (Swann et al., 2009). It more likely acts as a break, possibly to prevent automatic behavior or to slow down responses (Aron et al., 2014). Finally, prefrontal beta is not the only measure to investigate prefrontal processes with EEG, just recently prefrontal ERPs (pN, pP) being in association with motor inhibition have been reported (Berchicci et al., 2016; Di Russo et al., 2016). It might be that these reflect similar processes as prefrontal beta and this could be tested in future studies.

The most-studied ERP component of response inhibition is the stop-P3 (Eimer, 1993; Kopp et al., 1996; Bokura et al., 2001; Huster et al., 2013). P3 has been linked to cognitive control (Pires et al., 2014) and suggested to stem from PFC (Wessel et al., 2016). In the present study, we observed a higher P3 as correlate of both proactive and reactive motor inhibition. Interestingly, the P3 topographies in frequent and rare trials were different (Figure 5). In frequent trials, the enhanced P3 in No Stop- relative to Frequent Go-trials was centered on bilateral motor cortex, whereas the P3 effect in rare trials showed a broader, more central and posterior topography. The P3 effect in frequent trials might reflect proactive inhibitory influence directly on the premotor or motor cortex. The broader, more central P3 in rare trials could reflect reactive engagement of the preSMA (Albert et al., 2013). These effects however, have to be interpreted considering that we applied Laplace transformation (Kayser and Tenke, 2015). This method reduces the blurring effects of volume conduction on EEG data and increases spatial resolution (Burle et al., 2015). It enhances focal while reducing broad effects and minimizes the contribution of sources localized deep in the brain (Luck, 2014). Thus, claims about the topography and specific sources of the P3 effects, as well as comparisons with previous P3 findings have to be taken cautiously. Alternatively, the increased P3 in Rare Go-trials might reflect an enhanced motor preparation as the go-P3 has been proposed to be superimposed by motor-related potentials (Verleger et al., 2006). The observed P3 effects parallel the target-evoked prefrontal beta findings. For both P3 and beta power, trials where subjects had prepared to stop beforehand (MS) showed an enhanced amplitude relative to trials where they did not have to (CG). This suggests that during the implementation of inhibitory motor control, similar mechanisms play a role both when actually canceling the motor output as in Stop-trials and when transiently braking the motor execution to allow for more controlled response selection as in No Stop-trials.

## Conclusion

Our results shed light on the temporal dynamics of activity during proactive and reactive motor inhibition, including more generic (attention, cognitive control) and specific mechanisms (sensorimotor activity). As predicted by the DMC framework, activity in prefrontal, sensorimotor and visuoperceptual brain regions was modulated by proactive control. Prefrontal regions however, did not show sustained activity before target signals but only were activated transiently after target onset. Being prepared to stop resulted in enhanced attention for relevant visual signals, which was reflected in reduced occipital alpha power, an enhanced CNV and an increased target-evoked visual N1. At the same time, proactive motor control modulated activity in the sensorimotor cortex, particularly over ipsilateral sites. More precisely, if cues indicated that no nogo-signal was to be expected, beta power was enhanced over ipsilateral motor cortex, presumably facilitating response execution. When stopping was anticipated this facilitation was reduced, but no modulation of activity in the contralateral motor cortex was found. Finally, target-related actual implementation of response inhibition was associated with an enhanced P3 and increased prefrontal beta power. Both effects were observed also when participants were prepared to stop but eventually had to respond, which indicates that the same mechanisms are involved during response preparation when actually slowing down or when completely inhibiting response execution. However, these mechanisms were different from visuoperceptual and sensorimotor processes engaged proactively during the cue-target interval.

## Author Contributions

ML analyzed the data, interpreted the results and wrote the manuscript. IP recruited participants, collected the data and participated in data analysis. ET programmed the experiment and offered helpful knowledge for data analysis, interpretation and for writing the manuscript. UMK designed and supervised the study, interpreted the data and revised the manuscript. All authors approved the final version of the manuscript.

## Funding

This work was supported by intramural grants of the University of Lübeck (SPP-C4). We acknowledge financial support by Land Schleswig-Holstein within the funding programme Open Access Publikationsfonds.

## Supplementary Material

The Supplementary Material for this article can be found online at: http://journal.frontiersin.org/article/10.3389/fnhum.2017.00204/full#supplementary-material

## Conflict of Interest Statement

The authors declare that the research was conducted in the absence of any commercial or financial relationships that could be construed as a potential conflict of interest.

## References

[B1] Albert J. López-Martín S. Hinojosa J. A. Carretié L. (2013). Spatiotemporal characterization of response inhibition. Neuroimage 76, 272–281. 10.1016/j.neuroimage.2013.03.011 23523776

[B2] Alegre M. Gurtubay I. G. Labarga A. Iriarte J. Valencia M. Artieda J. (2004). Frontal and central oscillatory changes related to different aspects of the motor process: a study in go/no-go paradigms. Exp. Brain Res. 159, 14–22. 10.1007/s00221-004-1928-8 15480586

[B3] Aron A. R. (2011). From reactive to proactive and selective control: developing a richer model for stopping inappropriate responses. Biol. Psychiatry 69, e55–e68. 10.1016/j.biopsych.2010.07.024 20932513 PMC3039712

[B4] Aron A. R. Robbins T. W. Poldrack R. A. (2004). Inhibition and the right inferior frontal cortex. Trends Cogn. Sci. 8, 170–177. 10.1016/j.tics.2004.02.010 15050513

[B5] Aron A. R. Robbins T. W. Poldrack R. A. (2014). Inhibition and the right inferior frontal cortex: one decade on. Trends Cogn. Sci. 18, 177–185. 10.1016/j.tics.2013.12.003 24440116

[B7] Babiloni F. Babiloni C. Fattorini L. Carducci F. Onorati P. Urbano A. (1995). Performances of surface laplacian estimators: a study of simulated and real scalp potential distributions. Brain Topogr. 8, 35–45. 10.1007/bf01187668 8829389

[B6] Babiloni C. Brancucci A. Arendt-Nielsen L. Babiloni F. Capotosto P. Carducci F.. (2004). Alpha event-related desynchronization preceding a go/no-go task: a high-resolution EEG study. Neuropsychology 18, 719–728. 10.1037/0894-4105.18.4.719 15506840

[B8] Berchicci M. Spinelli D. Russo F. D. (2016). New insights into old waves. Matching stimulus- and response-locked ERPs on the same time-window. Biol. Psychol. 117, 202–215. 10.1016/j.biopsycho.2016.04.007 27086274

[B9] Boehm U. van Maanen L. Forstmann B. Van Rijn H. (2014). Trial-by-trial fluctuations in CNV amplitude reflect anticipatory adjustment of response caution. Neuroimage 96, 95–105. 10.1016/j.neuroimage.2014.03.063 24699015

[B10] Bokura H. Yamaguchi S. Kobayashi S. (2001). Electrophysiological correlates for response inhibition in a Go/NoGo task. Clin. Neurophysiol. 112, 2224–2232. 10.1016/s1388-2457(01)00691-5 11738192

[B11] Braver T. S. (2012). The variable nature of cognitive control: a dual mechanisms framework. Trends Cogn. Sci. 16, 106–113. 10.1016/j.tics.2011.12.010 22245618 PMC3289517

[B12] Braver T. S. Gray J. R. Burgess G. C. (2007). “Explaining the many varieties of working memory variation: dual mechanisms of cognitive control,” in Variation in Working Memory, eds Conway A. R. A. Jarrold C. Kane M. J. Miyake A. Towse J. N. (Oxford, UK: Oxford University Press), 76–106.

[B13] Brunia C. H. (1988). Movement and stimulus preceding negativity. Biol. Psychol. 26, 165–178. 10.1016/0301-0511(88)90018-x 3061478

[B14] Brunia C. H. Van Boxtel G. J. M. (2001). Wait and see. Int. J. Psychophysiol. 43, 59–75. 10.1016/S0167-8760(01)00179-9 11742685

[B15] Burle B. Spieser L. Roger C. Casini L. Hasbroucq T. Vidal F. (2015). Spatial and temporal resolutions of EEG: is it really black and white? A scalp current density view. Int. J. Psychophysiol. 97, 210–220. 10.1016/j.ijpsycho.2015.05.004 25979156 PMC4548479

[B16] Cheyne D. O. (2013). MEG studies of sensorimotor rhythms: a review. Exp. Neurol. 245, 27–39. 10.1016/j.expneurol.2012.08.030 22981841

[B17] Delorme A. Makeig S. (2004). EEGLAB: an open source toolbox for analysis of single-trial EEG dynamics including independent component analysis. J. Neurosci. Methods 134, 9–21. 10.1016/j.jneumeth.2003.10.009 15102499

[B18] Di Russo F. Lucci G. Sulpizio V. Berchicci M. Spinelli D. Pitzalis S.. (2016). Spatiotemporal brain mapping during preparation, perception, and action. Neuroimage 126, 1–14. 10.1016/j.neuroimage.2015.11.036 26608247

[B19] Eimer M. (1993). Effects of attention and stimulus probability on ERPs in a Go/Nogo task. Biol. Psychol. 35, 123–138. 10.1016/0301-0511(93)90009-w 8507742

[B20] Ergenoglu T. Demiralp T. Bayraktaroglu Z. Ergen M. Beydagi H. Uresin Y. (2004). Alpha rhythm of the EEG modulates visual detection performance in humans. Cogn. Brain Res. 20, 376–383. 10.1016/j.cogbrainres.2004.03.009 15268915

[B21] Filipović S. R. Jahanshahi M. Rothwell J. C. (2001). Uncoupling of contingent negative variation and alpha band event-related desynchronization in a go/no-go task. Clin. Neurophysiol. 112, 1307–1315. 10.1016/s1388-2457(01)00558-2 11516743

[B22] Fogelson N. Shah M. Scabini D. Knight R. T. (2009). Prefrontal cortex is critical for contextual processing: evidence from brain lesions. Brain 132, 3002–3010. 10.1093/brain/awp230 19713281 PMC2768662

[B23] Foxe J. J. Snyder A. C. (2011). The role of alpha-band brain oscillations as a sensory suppression mechanism during selective attention. Front. Psychol. 2:154. 10.3389/fpsyg.2011.00154 21779269 PMC3132683

[B24] Fuentemilla L. Cucurell D. Marco-Pallarés J. Guitart-Masip M. Morís J. Rodríguez-Fornells A. (2013). Electrophysiological correlates of anticipating improbable but desired events. Neuroimage 78, 135–144. 10.1016/j.neuroimage.2013.03.062 23583745

[B25] Hanslmayr S. Gross J. Klimesch W. Shapiro K. L. (2011). The role of α oscillations in temporal attention. Brain Res. Rev. 67, 331–343. 10.1016/j.brainresrev.2011.04.002 21592583

[B26] Hanslmayr S. Klimesch W. Sauseng P. Gruber W. Doppelmayr M. Freunberger R.. (2005). Visual discrimination performance is related to decreased alpha amplitude but increased phase locking. Neurosci. Lett. 375, 64–68. 10.1016/j.neulet.2004.10.092 15664124

[B27] Hillyard S. A. Anllo-Vento L. (1998). Event-related brain potentials in the study of visual selective attention. Proc. Natl. Acad. Sci. U S A 95, 781–787. 10.1073/pnas.95.3.781 9448241 PMC33798

[B28] Huster R. J. Enriquez-Geppert S. Lavallee C. F. Falkenstein M. Herrmann C. S. (2013). Electroencephalography of response inhibition tasks: functional networks and cognitive contributions. Int. J. Psychophysiol. 87, 217–233. 10.1016/j.ijpsycho.2012.08.001 22906815

[B29] Jahfari S. Verbruggen F. Frank M. J. Waldorp L. J. Colzato L. Ridderinkhof K. R.. (2012). How preparation changes the need for top-down control of the basal ganglia when inhibiting premature actions. J. Neurosci. 32, 10870–10878. 10.1523/JNEUROSCI.0902-12.2012 22875921 PMC6621019

[B30] Jung T.-P. Makeig S. Westerfield M. Townsend J. Courchesne E. Sejnowski T. J. (2000). Removal of eye activity artifacts from visual event-related potentials in normal and clinical subjects. Clin. Neurophysiol. 111, 1745–1758. 10.1016/s1388-2457(00)00386-2 11018488

[B31] Kajihara T. Anwar M. N. Kawasaki M. Mizuno Y. Nakazawa K. Kitajo K. (2015). Neural dynamics in motor preparation: from phase-mediated global computation to amplitude-mediated local computation. Neuroimage 118, 445–455. 10.1016/j.neuroimage.2015.05.032 26003857

[B32] Kayser J. (2009). Current source density (CSD) interpolation using spherical splines—CSD toolbox (Version 1.1). New York State Psychiatric Institute: Division of Cognitive Neuroscience Available online at: http://psychophysiology.cpmc.columbia.edu/Software/CSDtoolbox

[B33] Kayser J. Tenke C. E. (2006). Principal components analysis of Laplacian waveforms as a generic method for identifying ERP generator patterns: I. Evaluation with auditory oddball tasks. Clin. Neurophysiol. 117, 348–368. 10.1016/j.clinph.2005.08.034 16356767

[B34] Kayser J. Tenke C. E. (2015). On the benefits of using surface Laplacian (current source density) methodology in electrophysiology. Int. J. Psychophysiol. 97, 171–173. 10.1016/j.ijpsycho.2015.06.001 26071227 PMC4610715

[B35] Klimesch W. (2012). α-band oscillations, attention, and controlled access to stored information. Trends Cogn. Sci. 16, 606–617. 10.1016/j.tics.2012.10.007 23141428 PMC3507158

[B36] Kopp B. Mattler U. Goertz R. Rist F. (1996). N2, P3 and the lateralized readiness potential in a nogo task involving selective response priming. Electroencephalogr. Clin. Neurophysiol. 99, 19–27. 10.1016/0921-884x(96)95617-9 8758967

[B37] Krämer U. M. Knight R. T. Münte T. F. (2011). Electrophysiological evidence for different inhibitory mechanisms when stopping or changing a planned response. J. Cogn. Neurosci. 23, 2481–2493. 10.1162/jocn.2010.21573 20849230

[B38] Krämer U. M. Solbakk A. K. Funderud I. Løvstad M. Endestad T. Knight R. T. (2013). The role of the lateral prefrontal cortex in inhibitory motor control. Cortex 49, 837–849. 10.1016/j.cortex.2012.05.003 22699024 PMC3443703

[B39] Kroeger J. Bäumer T. Jonas M. Rothwell J. C. Siebner H. R. Munchau A. (2010). Charting the excitability of premotor to motor connections while withholding or initiating a selected movement. Eur. J. Neurosci. 32, 1771–1779. 10.1111/j.1460-9568.2010.07442.x 21059111

[B40] Lavallee C. F. Meemken M. T. Herrmann C. S. Huster R. J. (2014). When holding your horses meets the deer in the headlights: time-frequency characteristics of global and selective stopping under conditions of proactive and reactive control. Front. Hum. Neurosci. 8:994. 10.3389/fnhum.2014.00994 25540615 PMC4262052

[B41] Lopez-Calderon J. Luck S. J. (2014). ERPLAB: an open-source toolbox for the analysis of event-related potentials. Front. Hum. Neurosci. 8:213. 10.3389/fnhum.2014.00213 24782741 PMC3995046

[B42] Luck S. J. (2014). An Introduction to the Event-Related Potential Technique. Cambridge, MA: MIT press.

[B43] Luck S. J. Woodman G. F. Vogel E. K. (2000). Event-related potential studies of attention. Trends Cogn. Sci. 4, 432–440. 10.1016/S1364-6613(00)01545-X 11058821

[B44] Neuper C. Wörtz M. Pfurtscheller G. (2006). ERD/ERS patterns reflecting sensorimotor activation and deactivation. Prog. Brain Res. 159, 211–222. 10.1016/s0079-6123(06)59014-4 17071233

[B45] Nuwer M. R. Comi G. Emerson R. Fuglsang-Frederiksen A. Guérit J. M. Hinrichs H.. (1998). IFCN standards for digital recording of clinical EEG. Electroencephalogr. Clin. Neurophysiol. 106, 259–261. 10.1016/s0013-4694(97)00106-5 9743285

[B46] Picazio S. Veniero D. Ponzo V. Caltagirone C. Gross J. Thut G.. (2014). Prefrontal control over motor cortex cycles at beta frequency during movement inhibition. Curr. Biol. 24, 2940–2945. 10.1016/j.cub.2014.10.043 25484293 PMC4274313

[B47] Pires L. Leitão J. Guerrini C. Simões M. R. (2014). Event-related brain potentials in the study of inhibition: cognitive control, source localization and age-related modulations. Neuropsychol. Rev. 24, 461–490. 10.1007/s11065-014-9275-4 25407470

[B48] Romei V. Gross J. Thut G. (2010). On the role of prestimulus alpha rhythms over occipito-parietal areas in visual input regulation: correlation or causation? J. Neurosci. 30, 8692–8697. 10.1523/JNEUROSCI.0160-10.2010 20573914 PMC6634639

[B49] Salmelin R. Hari R. (1994). Spatiotemporal characteristics of sensorimotor neuromagnetic rhythms related to thumb movement. Neuroscience 60, 537–550. 10.1016/0306-4522(94)90263-1 8072694

[B50] Schevernels H. Bombeke K. Krebs R. M. Boehler C. N. (2016). Preparing for (valenced) action: the role of differential effort in the orthogonalized go/no-go task. Psychophysiology 53, 186–197. 10.1111/psyp.12558 26481327

[B51] Smith J. L. Barry R. J. Steiner G. Z. (2013). CNV resolution does not cause NoGo anteriorisation of the P3: a failure to replicate Simson et al. Int. J. Psychophysiol. 89, 349–357. 10.1016/j.ijpsycho.2013.05.002 23669175

[B52] Smith J. L. Johnstone S. J. Barry R. J. (2008). Movement-related potentials in the Go/NoGo task: the P3 reflects both cognitive and motor inhibition. Clin. Neurophysiol. 119, 704–714. 10.1016/j.clinph.2007.11.042 18164657

[B53] Solbakk A. K. Funderud I. Løvstad M. Endestad T. Meling T. Lindgren M.. (2014). Impact of orbitofrontal lesions on electrophysiological signals in a stop signal task. J. Cogn. Neurosci. 26, 1528–1545. 10.1162/jocn_a_00561 24392904 PMC4090109

[B54] Swann N. C. Cai W. Conner C. R. Pieters T. A. Claffey M. P. George J. S.. (2012). Roles for the pre-supplementary motor area and the right inferior frontal gyrus in stopping action: electrophysiological responses and functional and structural connectivity. Neuroimage 59, 2860–2870. 10.1016/j.neuroimage.2011.09.049 21979383 PMC3322194

[B55] Swann N. C. Poizner H. Houser M. Gould S. Greenhouse I. Cai W.. (2011). Deep brain stimulation of the subthalamic nucleus alters the cortical profile of response inhibition in the beta frequency band: a scalp EEG study in Parkinson’s disease. J. Neurosci. 31, 5721–5729. 10.1523/JNEUROSCI.6135-10.2011 21490213 PMC3086079

[B56] Swann N. C. Tandon N. Canolty R. Ellmore T. M. Mcevoy L. K. Dreyer S.. (2009). Intracranial EEG reveals a time- and frequency-specific role for the right inferior frontal gyrus and primary motor cortex in stopping initiated responses. J. Neurosci. 29, 12675–12685. 10.1523/JNEUROSCI.3359-09.2009 19812342 PMC2801605

[B57] Swann N. C. Tandon N. Pieters T. A. Aron A. R. (2013). Intracranial electroencephalography reveals different temporal profiles for dorsal- and ventro-lateral prefrontal cortex in preparing to stop action. Cereb. Cortex 23, 2479–2488. 10.1093/cercor/bhs245 22879352 PMC3767964

[B58] Taniguchi M. Kato A. Fujita N. Hirata M. Tanaka H. Kihara T.. (2000). Movement-related desynchronization of the cerebral cortex studied with spatially filtered magnetoencephalography. Neuroimage 12, 298–306. 10.1006/nimg.2000.0611 10944412

[B59] Tecce J. J. (1972). Contingent negative variation (CNV) and psychological processes in man. Psychol. Bull. 77, 73–108. 10.1037/h0032177 4621420

[B60] Teolis A. (1998). Computational Signal Processing with Wavelets. Berlin: Springer Science+Business Media.

[B61] Tzagarakis C. West S. Pellizzer G. (2015). Brain oscillatory activity during motor preparation: effect of directional uncertainty on beta, but not alpha, frequency band. Front. Neurosci. 9:246. 10.3389/fnins.2015.00246 26257597 PMC4508519

[B62] Verbruggen F. Logan G. D. (2008). Response inhibition in the stop-signal paradigm. Trends Cogn. Sci. 12, 418–424. 10.1016/j.tics.2008.07.005 18799345 PMC2709177

[B63] Verbruggen F. Stevens T. Chambers C. D. (2014). Proactive and reactive stopping when distracted: an attentional account. J. Exp. Psychol. Hum. Percept. Perform. 40, 1295–1300. 10.1037/a0036542 24842070 PMC4120704

[B64] Verleger R. Paehge T. Kolev V. Yordanova J. Jaskowski P. (2006). On the relation of movement-related potentials to the go/no-go effect on P3. Biol. Psychol. 73, 298–313. 10.1016/j.biopsycho.2006.05.005 16837117

[B65] Vink M. Kaldewaij R. Zandbelt B. B. Pas P. Du Plessis S. (2015). The role of stop-signal probability and expectation in proactive inhibition. Eur. J. Neurosci. 41, 1086–1094. 10.1111/ejn.12879 25832122

[B66] Wessel J. R. Aron A. R. (2015). It’s not too late: the onset of the frontocentral P3 indexes successful response inhibition in the stop-signal paradigm. Psychophysiology 52, 472–480. 10.1111/psyp.12374 25348645 PMC4830357

[B67] Wessel J. R. Ullsperger M. Obrig H. Villringer A. Quinque E. Schroeter M. L.. (2016). Neural synchrony indexes impaired motor slowing after errors and novelty following white matter damage. Neurobiol. Aging 38, 205–213. 10.1016/j.neurobiolaging.2015.10.014 26563990

[B68] Zaepffel M. Trachel R. Kilavik B. E. Brochier T. (2013). Modulations of EEG beta power during planning and execution of grasping movements. PLoS One 8:e60060. 10.1371/journal.pone.0060060 23555884 PMC3605373

[B69] Zandbelt B. B. Bloemendaal M. Neggers S. F. Kahn R. S. Vink M. (2013). Expectations and violations: delineating the neural network of proactive inhibitory control. Hum. Brain Mapp. 34, 2015–2024. 10.1002/hbm.22047 22359406 PMC6869973

[B70] Zumer J. M. Scheeringa R. Schoffelen J. M. Norris D. G. Jensen O. (2014). Occipital alpha activity during stimulus processing gates the information flow to object-selective cortex. PLoS Biol. 12:e1001965. 10.1371/journal.pbio.1001965 25333286 PMC4205112

