# Supplementary material for: Temporal Dynamics of Proactive and Reactive Motor Inhibition
Source: Front Hum Neurosci. 2017 Apr 27;11:204. doi: 10.3389/fnhum.2017.00204 (PMC5406465; doi:10.3389/fnhum.2017.00204)
Supplement: Supplementary file 1 [file Image_1.pdf]

Supplementary Material

Temporal Dynamics of Proactive and Reactive Motor Inhibition

Matthias Liebrand, Inga Pein, Elinor Tzvi and Ulrike M. Krämer

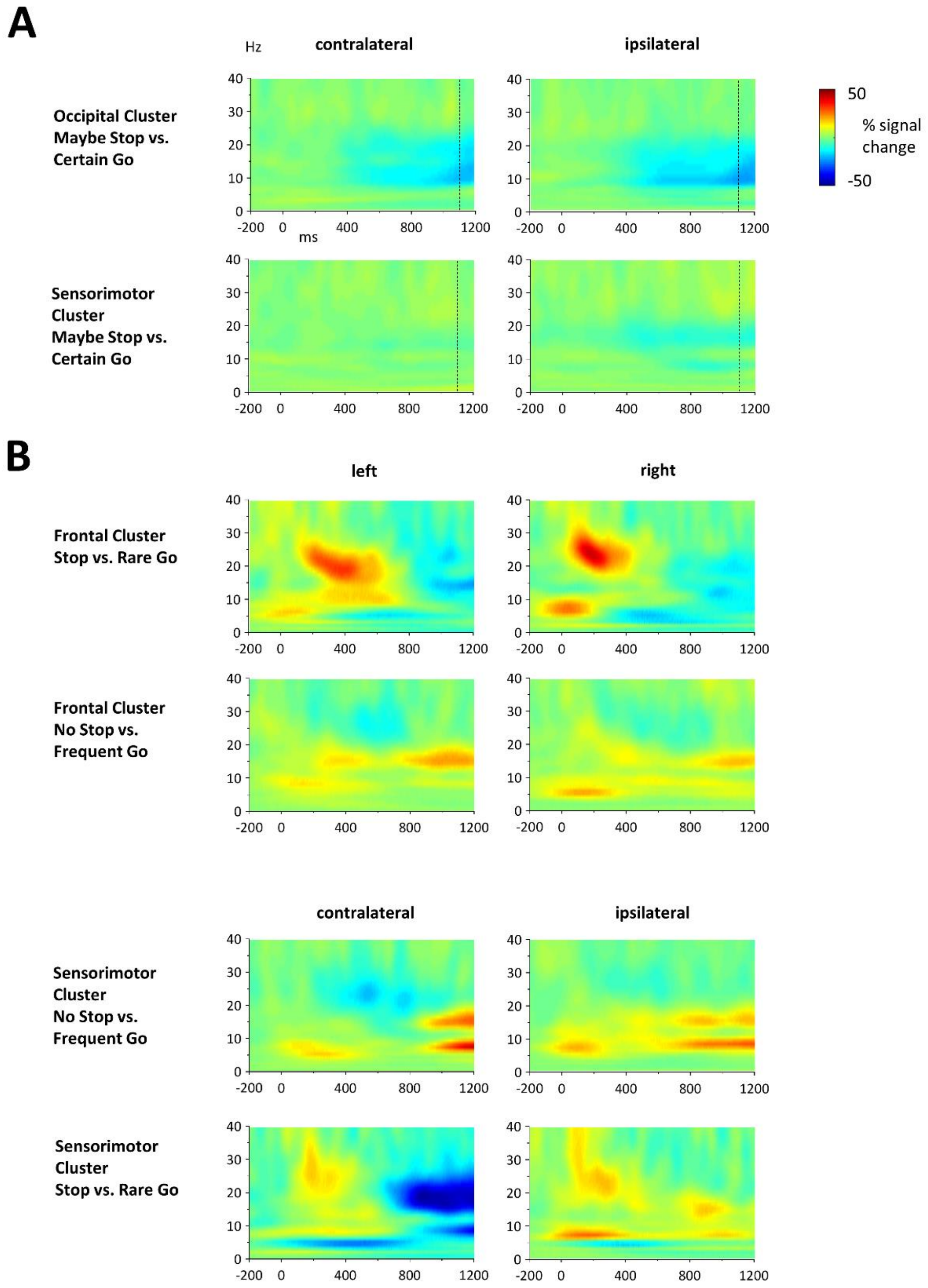

Supplementary Figure S1 | Time-frequency plots. In (A) data of the cue-target interval is displayed and in (B) target-related effects. Time-frequency plots are presented as differences between conditions at certain clusters (frontal/sensorimotor/occipital). In the cue-target interval at 1100 ms the target appeared. Note that data at sensorimotor clusters is flipped between hemispheres.

SUPPLEMENTARY RESULTS

Activity in the gamma band

As suggested by one reviewer, additionally to alpha/mu and beta power we investigated gamma activity (31-40 Hz). To examine gamma power the same clusters were used as for alpha/mu and beta activity. In the cue-target interval (700-1100 ms) there was no difference in frontal gamma between Maybe Stop- and Certain Go-trials ($F_{1,21} = 1.9$, $p = 0.178$). As target related effect (200-500 ms) frontal gamma did not differ between No Stop- and Frequent Go-trials ($F_{1,21} = 3.0$, $p = 0.1$). Frontal gamma tended (200-500 ms) to be increased in Stop- compared to Rare Go-trials ($F_{1,21} = 4.2$, $p = 0.053$). This latter finding supports our results obtained in the beta band.

Regarding gamma over occipital electrodes, in the cue-target interval (700-1100 ms) there was also no difference between Maybe Stop- and Certain Go-trials ($F_{1,21} = 0.1$, $p = 0.708$). As target related effects (200-500 ms) neither No Stop- and Frequent Go-trials ($F_{1,21} = 2.9$, $p = 0.102$) nor Stop- and Rare Go-trials ($F_{1,21} = 1.8$, $p = 0.190$) did show differences in occipital gamma.
